# SIRT1: A Key Player in Male Reproduction

**DOI:** 10.3390/life12020318

**Published:** 2022-02-21

**Authors:** Muhammad Babar Khawar, Abdullah Muhammad Sohail, Wei Li

**Affiliations:** 1 Guangzhou Women and Children’s Medical Center, Guangzhou Medical University, Guangzhou 510623, China; 2 Applied Molecular Biology and Biomedicine Laboratory, Department of Zoology, University of Narowal, Narowal 51600, Pakistan; 3 State Key Laboratory of Stem Cell and Reproductive Biology, Institute of Zoology, Chinese Academy of Sciences, Beijing 100101, China; 4 Molecular Medicine and Cancer Therapeutics Laboratory, Department of Zoology, Faculty of Sciences, University of Central Punjab, Lahore 54782, Pakistan

**Keywords:** sirtuins, SIRT1, spermatogenesis, steroidogenesis, reproductive medicine

## Abstract

Reproduction is the way to immortality for an individual, and it is essential to the continuation of the species. Sirtuins are involved in cellular homeostasis, energy metabolism, apoptosis, age-related problems, and sexual reproduction. Sirtuin 1 (SIRT1) belongs to the sirtuin family of deacetylases, and it is a nicotinamide adenine dinucleotide (NAD)-dependent deacetylase. It removes the acetyl group from a variety of substrates. SIRT1 regulates endocrine/metabolic, reproductive, and placental development by deacetylating histone, different transcription factors, and signal transduction molecules in a variety of cellular processes. It also plays a very important role in the synthesis and secretion of sex hormones via regulating the hypothalamus-pituitary-gonadal (HPG) axis. Moreover, SIRT1 participates in several key stages of spermatogenesis and sperm maturation. The current review will give a thorough overview of SIRT1’s functions in male reproductive processes, thus paving the way for more research on restorative techniques and their uses in reproductive medicine.

## 1. Introduction

Sirtuins, also known as the Sir2 family, encode seven distinct sirtuins in mammals that are referred to as sirtuin 1 (SIRT1)-SIRT7 [1]. In somatic cells, sirtuins are present in several places; for example, SIRT1, SIRT6, and SIRT7 are found in the nucleus, whereas SIRT4 and SIRT5 are found in the mitochondria. SIRT2 and SIRT3 are mostly localized in the cytoplasm and mitochondria, but they migrate to the nucleus in response to stress and during the G2/M cell cycle transition [2]. Sirtuins are cellular stress sensors that regulate mitochondrial and nuclear activity to help cells adapt to stressful situations. Sirtuins perform the deacetylation and ADP-ribosylation of numerous substrates in an nicotinamide adenine dinucleotide (NAD^+^ (cofactor)) dependent manner [3]. Sirtuins can be triggered in a variety of ways, including the NAD^+^/NADH ratio or the activation of kinases such as AMP-activated protein kinase and c-Jun N-terminal kinase 1 [2,4]. Nuclear sirtuins control the basic aspects of genome biology through epigenetic modifications or regulating key transcription and other chromatin factors, whereas mitochondrial sirtuins control the activity of major metabolic and reactive oxygen species (ROS)-detoxification enzymes. The lifespan and aging are also controlled by sirtuins [5,6], and overexpression of SIRT1 and SIRT6 have been shown to promote longevity [7,8]. Many early age-related problems were detected in various sirtuins family member knockout models [9,10], and SIRT2, SIRT3, and SIRT4 deficiency were linked to a higher risk of cancer [11,12,13]. Extensive research on the expression of sirtuins in a variety of pathological circumstances suggests that regulating their activity may aid in the treatment of certain metabolic, neurological, and cancer disorders, as well as diseases associated with oxidative stress. Sirtuins may be used as prognostic markers for some pathological conditions and may be useful in assessing their development, particularly by modifying their activity, due to their broad range of activities [14]. The expression of sirtuins in mammalian testes triggers the curiosity of reproductive biologists, who want to learn more about their possible involvement in reproduction [15].

## 2. Sirtuin 1 (SIRT1)

SIRT1 is the most conserved member of the sirtuin family, and it is analogous to yeast Sir2 [16]. SIRT1 is a NAD^+^-dependent class III deacetylase, performing numerous functions from metabolism to apoptosis [17]. SIRT1 is expressed in several tissues including the liver and gonads [18,19,20,21]. SIRT1 is normally found in the nucleus, but in the case of physiological stress or pathological states, it can be translocated into the cytoplasm, making it a unique case among the sirtuin family members [22]. SIRT1 regulates the physiological activity of several epigenetic and non-epigenetic targets by removing the functional acetyl group from histone proteins, transcription factors, and co-factors [23]. SIRT1 regulates several downstream pathways including lipid/glucose metabolism, inflammatory pathways, differentiation, cell death, and autophagy via controlling the acetylation of some substrates [24,25,26].

SIRT1 is referred to as a nutrient sensor due to its deacetylase activity, which is determined by the proportion of NAD^+^/NADH. The role of SIRT1 in regulating the activity of liver and fat tissues is well documented [27]. SIRT1 tends to reduce fat storage and protects adipose tissue from obesity-induced inflammation [28,29,30]; it controls fatty acid metabolism and increases oxidative metabolism in the liver [31,32]; and it improves glucose tolerance by regulating insulin synthesis in the pancreas [33,34]. Fasting triggers SIRT1 activity to enhance gluconeogenesis and fatty acid oxidation consequently lowering adipogenesis and insulin production, and excessive consumption leads to reduced activity of SIRT1 in vertebrates [27]. Although SIRT1 has been extensively studied, its role in male reproduction has only been explored recently. In the next section, we will review the current progress in understanding the significant roles played by SIRT1 in male reproductive processes.

## 3. SIRT1 in Male Germ Cells

**Figure 1:**
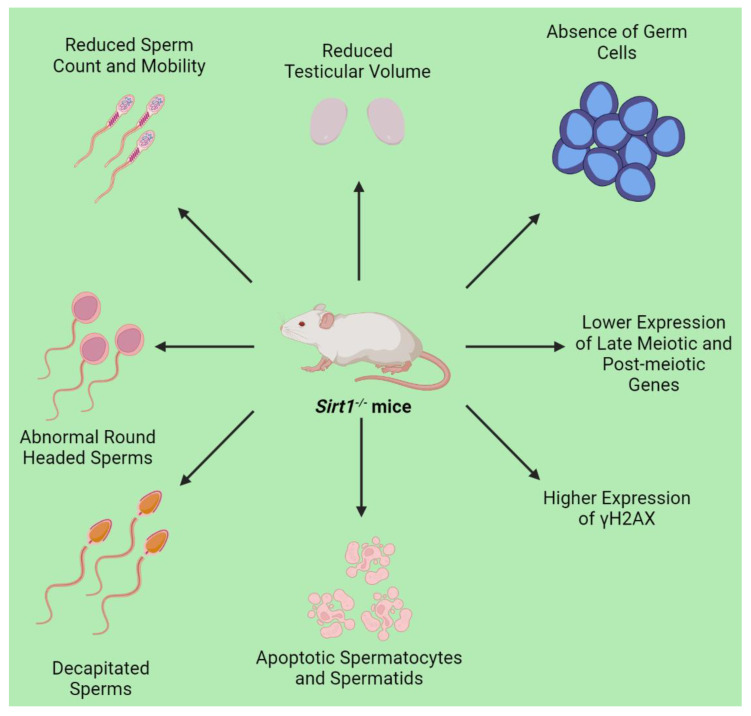
**The functional roles of SIRT1 in male reproductive processes.***Sirt1^−/−^* mice showed a range of phenotypic characteristics including reduced testis, decreased sperm counts, reduced motility, presence of abnormal spermatozoa, increase in the number of decapitated sperms, absence of germ cells, and increased apoptotic spermatocytes and spermatids. In addition, there was a higher γH2AX expression and significantly reduced expression of several key late meiotic and post-meiotic genes.

The sirtuin family member that has received the most interest is SIRT1. Immunoblotting results revealed that SIRT1 is expressed at a higher level in mouse testis. Immunohistochemistry has also revealed that SIRT1 is present at various stages of spermatogenesis [35]. The SIRT1 protein was found in the nuclei of spermatogenic cells of the testis. During germ cell development, type A spermatogonia had a weak, finely speckled nuclear staining, but type B and intermediate spermatogonia had a somewhat clear nuclear staining with coarser positive spots [36]. With diffusely speckled nuclear staining, spermatocytes and round spermatids were highly positive. The nuclear staining faded to barely a few spots shortly after the spermatids began to lengthen (step 9), and, by step 11, it was completely gone. This staining pattern shows that the SIRT1 protein is nuclear in late spermatogonia, spermatocytes, and round spermatids, with the highest in pachytene spermatocytes [36]. The presence of SIRT1 in the nuclei of spermatogenic cells is suggestive of its role in the development of germ cells during spermatogenesis [36]. The direct hint of the likely role of sirtuins in regulating male reproductive functions was observed in *Sirt1^−/−^* mice [36]. Though most of these mice did not survive due to defects in several vital organs, some of the survivors allowed the researchers to investigate the effects of SIRT1 deficiency on male reproduction [36,37]. SIRT1-deficient male mice suffer from infertility characterized by poor spermatogenesis and abnormal sperm maturation. The male reproductive gonads of *Sirt1*-deficient mice were found to be relatively smaller (Figure 1). Moreover, sperm counts and motility were significantly reduced in SIRT1-deficient mice (Figure 1). Similarly, abnormal sperms characterized by smaller and round heads as well as spermatozoa heads with detached tails were abundant. Among the anomalies, the absence of germ cells or the abundance of apoptotic spermatocytes and spermatids were frequently observed (Figure 1) [36]. In addition, a point mutation (H355Y) produced in the *Sirt1* gene inhibited the catalytic activity of SIRT1 protein in embryonic stem cells, which was subsequently used to create chimeric mice, and the homozygous mice with mutant allele also exhibited abnormal spermatogenesis. These mice had abnormal spermatogenesis characterized by lower immobile sperms and more apoptotic cells [38]. Thus, these findings infer that the catalytic activity of the SIRT1 protein is responsible for spermatogenesis.

### 3.1. Mechanism Underlying SIRT1-Related Germ Cell Death

**Figure 2:**
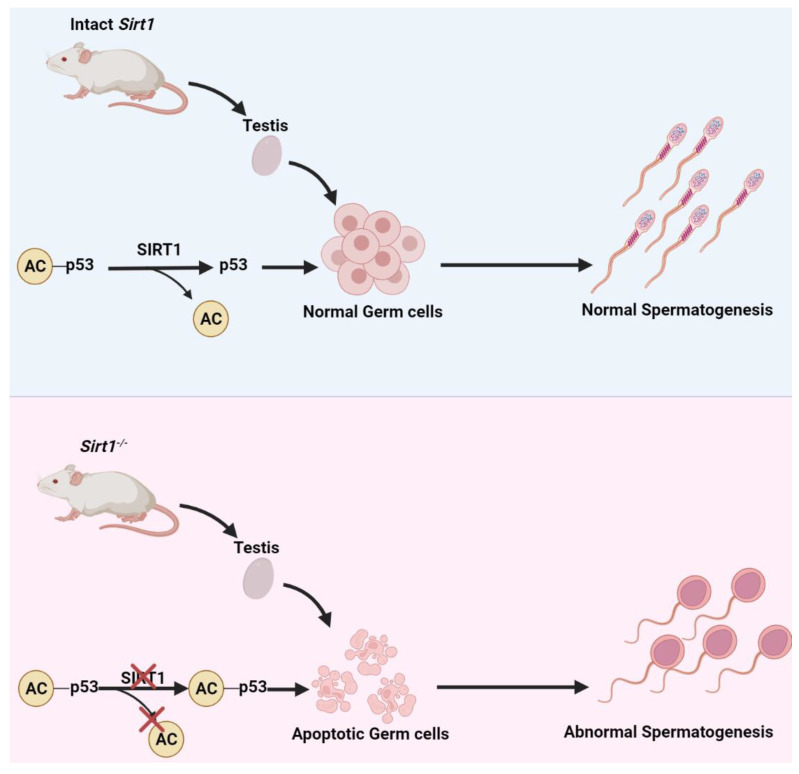
**SIRT1-mediated p53 deacetylation prevents germ cell death.** SIRT1 performs the deacetylation of p53 and renders it to induce apoptosis in germ cells. While in the absence of SIRT1 condition, acetylated p53 triggers germ cell apoptosis and infertility in *Sirt1^−/−^* mice.

A huge number of investigations have been undertaken in the search for molecular pathways by which SIRT1 deficiency results in spermatogenic aberration. A significant decrease in spermatogenic cells was found in earlier stages when crossed a *Sirt1*^−/−^ with an Oct4-GFP transgenic strain [37], and the *Sirt1*-deficient testes showed increased apoptosis in male germ cells and other testicular cells [37]. Male germ cell death in *Sirt1^−/−^* mice was related to a higher p53 activity, as testicular apoptosis is dependent on acetylation mediated-p53 activity (Figure 2) [36,39,40,41]. Moreover, *Sirt1^−/−^* mice had their genomic integrity distorted characterized by DNA damages [35,42]. A histological examination of the testis of *Sirt1^−/−^* mice signified its role in late meiotic stages and spermiogenesis characterized by a higher expression of γH2AX and lower expression of the late-meiotic and post-meiotic genes including *Polk*, *Prm1*, and *Prm2* (Figure 1). Since the Gene Ontology (GO) has shown several genes involved in spermatogenesis are differentially expressed in the testis of *Sirt1^−/−^* mice, SIRT1 deficiency might influence fertility by regulating the transcription of several spermatogenic genes [37]. In addition, *Sirt1^−/−^* mice had an aberrant expression of sumoylation-related genes, according to a microarray investigation of global testicular gene expression [37]. SIRT1-mediated deacetylation can impact sumoylation [43,44] that regulates numerous cellular physiological activities [45]. And sumoylation has been linked to many functions in testis and spermatogenesis, including germ cell proliferation, heterochromatin remodeling, and change in nuclear morphology [46,47,48,49,50,51].

**Figure 3:**
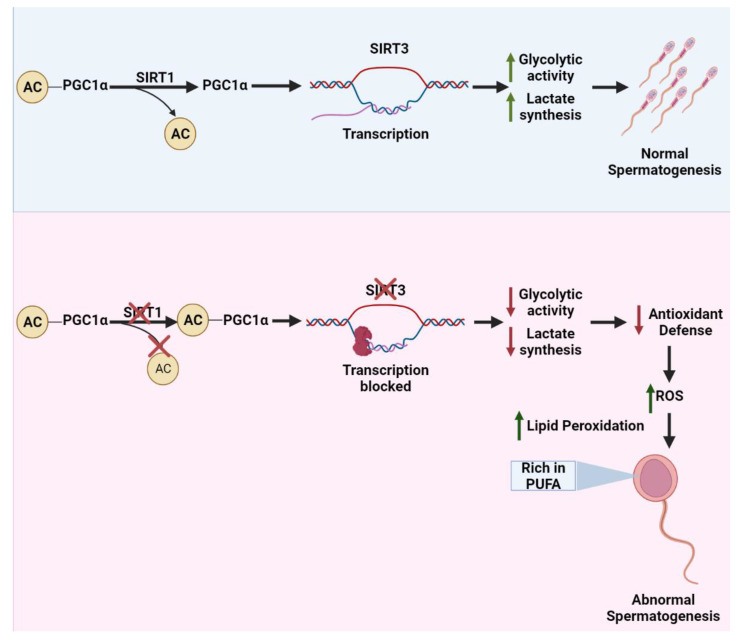
**SIRT1/PGC1α/SIRT3 axis regulates the glucose metabolism to support spermatogenesis.** SIRT1 deacetylates the PGC1α to promote the transcription of SIRT3 in the testis that ultimately enhances the antioxidant defense systems, glycolytic activity, and lactate synthesis which are required to ensure normal spermatogenesis. In *Sirt1^−/^^−^* mice, acetylated PGC1α fails to promote *Sirt3* transcription, decreasing glucose metabolism and antioxidant defense systems. As a result, the levels of ROS are elevated consequently, inducing the lipid peroxidation of polyunsaturated fatty acids-rich membranes of the sperms.

Moreover, SIRT1 along with SIRT3 and peroxisome proliferator-activated receptor γ coactivator 1 alpha (PGC1α) activate antioxidant defense systems, hence abnormal spermatogenesis in *Sirt1^−/−^* mice might be due to oxidative stress. Upon SIRT1-mediated deacetylation and activation, PGC1α stimulates the transcription of the *Sirt3* gene [52], which is also expressed in mammalian testicular tissue [15]. The relationship between SIRT1/PGC1α/SIRT3 dysregulation and reactive oxygen species (ROS)/antioxidant defense system was investigated in a pre-diabetic rat model [53]. Reduced SIRT3 levels were observed to enhance glycolysis in the rat testis, suggesting the involvement of sirtuins in the regulation of testis metabolism [53,54], which is consistent with the previous findings where reduced SIRT1 or SIRT3 expression was found to be associated with higher glycolytic activity in numerous tissues [55]. Since glucose metabolism and lactate synthesis are necessary for optimal spermatogenesis (Figure 3) [56,57], excessive glycolytic activity may lead to mitochondrial ROS overproduction [54]. Moreover, loss of SIRT1 or SIRT3 causes a malfunctioning electron transport chain, and a reduction in the activity of antioxidant defenses [58]. Because sperm membranes contain a large proportion of polyunsaturated fatty acids (PUFA), they are extremely vulnerable to oxidative stress [59]. The fluidity and fusogenicity are required for acrosomal processes and sperm–oolemma interactions are controlled by PUFAs. They are also, unfortunately, highly susceptible to lipid peroxidation (Figure 3) [60,61,62]. SIRT1 suppression and germ cell apoptosis might be regulated by oxidative stress as ROS can affect mitochondrial apoptotic pathways in several aspects [63]. Consistent with the above findings, long-term Bisphenol A (BPA) exposure decreases SIRT1 activity while increasing p53 acetylation, ROS, and DNA damage, thus affecting the late meiotic and post-meiotic spermatogenic stages [18].

### 3.2. SIRT1 in Acrosome Biogenesis

**Figure 4:**
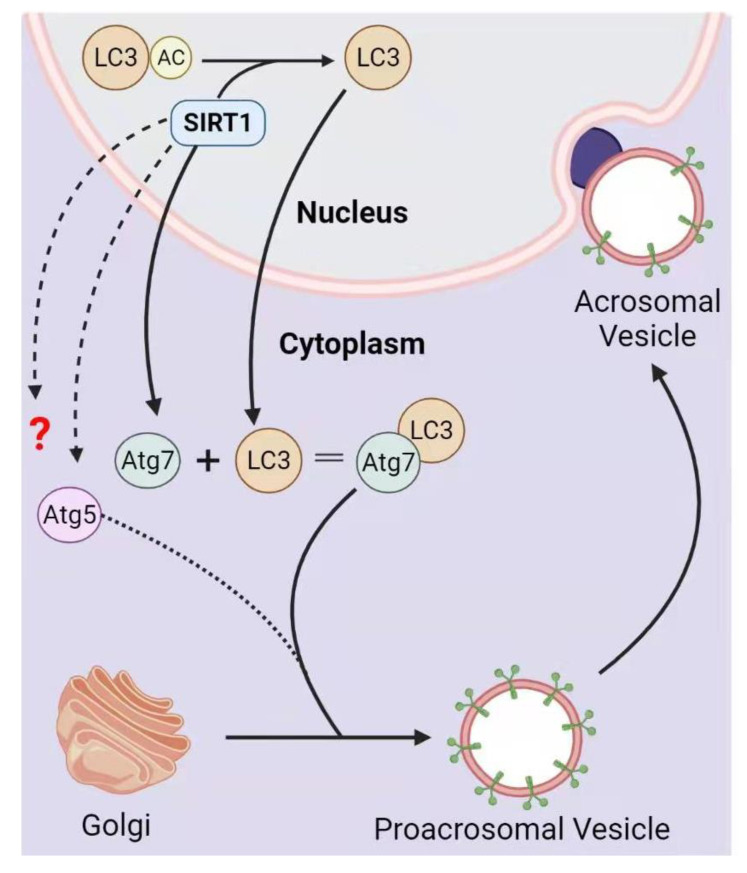
**The functional role of SIRT1 in acrosome biogenesis.** SIRT1 deacetylates the LC3 in the nucleus, which moves to the cytoplasm to interact with other components of autophagic machinery. The resulting complex of ATG7, LC3, and a few other deacetylated components guide the fusion of Golgi-derived proacrosomal vesicles to the acrosome, thus promoting acrosome biogenesis.

To study the germ cell-specific functional role of SIRT1, *Sirt^F/F^* mice were crossed with *Stra8-iCre* or *Prm1-Cre* mice to obtain germ cell-specific *Sirt1*-KO mice that lack exon 4 in pre-meiotic or post-meiotic germ cells, respectively. They found that germ cell-specific *Sirt1*-KO causes several abnormalities including a reduction in the number of sperms, histone modifications, protamine transition, chromatin remodeling, and abnormal sperms, collectively leading to male reproductive senescence [35,64]. We also generated germ cell-specific *Sirt1* knockout mice by crossing *Sirt^F/F^* mice with *Tnap-Cre*, which produced subfertile males when mated with wild-type females. We found significantly decreased sperm counts and abnormal spermatozoa with reduced sperm motility in these mice. Moreover, the immunofluorescence staining of their acrosomes with sperm protein 56 (sp56) revealed deformed, fragmented mislocalized acrosomes. Further investigation showed that the autophagic molecular marker microtubule-associated protein light chain 3 (LC3) was accumulated in the nucleus, which influences proacrosomal granule fusion to the nuclear membrane to form an acrosomic vesicle. Furthermore, we demonstrated that SIRT1 performs the deacetylation of LC3, which subsequently moves to the cytoplasm where autophagy-related gene 7 (ATG7) activates it, thus promoting autophagy-mediated acrosome biogenesis (Figure 4) [65]. In support of the above discovery, the levels of seminal SIRT1 in oligoasthenoteratozoospermic men were found to be notably decreased, and those suffering from varicocele have even lower SIRT1 levels, further supporting its role in acrosome biogenesis [66].

### 3.3. SIRT1-Mediated Protamine Replacement

During spermiogenesis, the histone-based nucleosomes must be removed and replaced by protamines that span most of the genome. Male mice were infertile when protamines were lost or when the testis-specific version of histone 2B was mutated because all of them affect histone removal [67,68]. Histone removal is triggered by nucleosome post-translational modifications. The testis-specific bromodomain protein BRDT binds to nucleosomes due to acetylation of histone H4 on sites K5 (H4K5) and K8 (H4K8) [69]. Transition protein 2 (TP2) and protamines (PRM) were unable to appropriately localize within the nuclei of elongating and condensing spermatids in the absence of BRDT-H4 interaction, resulting in aberrant chromatin condensation and finally infertility [70]. Recently, Bell et al. reported a chromatin condensation defect in *Sirt1-*deficient elongating and elongated spermatids, where TP2 failed to localize in the nucleus, and there was reduced acetylation of H4K5, H4K8, and H4K12 [35]. Similarly, abnormal histone to protamine transition and chromatin remodeling defects were observed in germ cell-specific *Sirt1*-knockout mice [35]; this defect makes sperm DNA more vulnerable to apoptotic/oxidative damage [71]. SIRT1 might also work in coordination with SIRT6 for chromatin regulation, as there was a downregulation and in turn poor sperm protamination in an obese mice model [72]. Collectively, SIRT1 might balance other factors to enhance H4 acetylation and the histone-to-protamine transition.

## 4. SIRT1 Functions in the Hypothalamic-Pituitary-Gonadal (HPG) Axis

**Figure 5:**
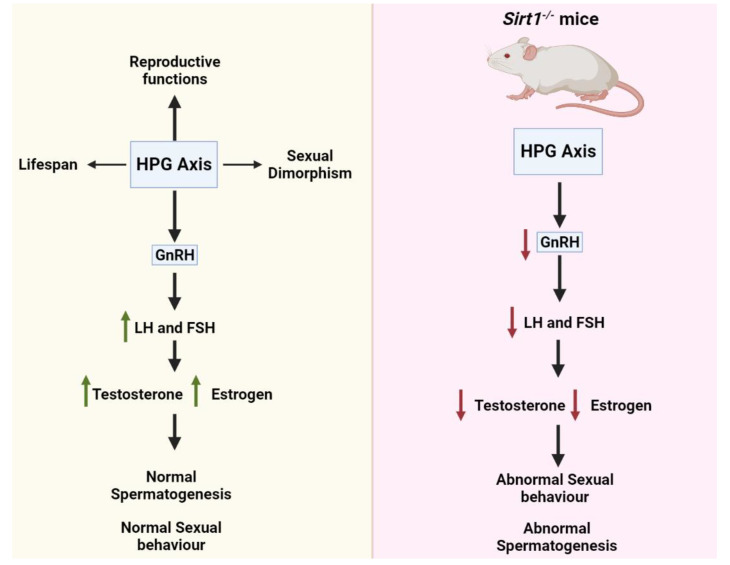
**The functional role of SIRT1 in the hypothalamus-pituitary-gonadal axis.** SIRT1 regulates the activity of the hypothalamus-pituitary-gonadal axis by controlling the release of GnRH. In the absence of SIRT1, the HPG axis fails to secrete more GnRH resulting in reduced LH and FSH secretion, consequently retarding the production of testosterone and estrogen. This reduction in sex hormones results in compromised sexual behavior and aberrant spermatogenesis.

The HPG axis plays a significant role in regulating reproductive functions, life cycle, and sexual dimorphism. SIRT1 is a key player in regulating the activities of the HPG (hypothalamus-pituitary–gonadal) axis and neuroendocrine systems (Figure 5) [73]. *Sirt1* is expressed in neurons as well, particularly those that control the hypothalamus’ metabolic activity [73]. There exists a diffused network of GnRH neurons called pulse-generator in the hypothalamus; it is responsible for the releasing of GnRH. Gonads synthesize estrogen and testosterone under the influence of LH and FSH secreted under the stimulation of pulsatile secretion of GnRH (Figure 5) [74,75]. *Sirt1*-knockout results in decreased hypothalamic gonadotropin-releasing hormone (GnRH) expression, and consequently lower serum LH and FSH levels and aberrant spermatogenesis, suggesting the significance of SIRT1 in regulating the HPG axis (Figure 5) [42]. Furthermore, *miR-132/212*-mediated action of GnRH involved a posttranscriptional decrease in *Sirt1*. Subsequently, SIRT1-dependent FOXO1 deacetylation was decreased, limiting FOXO1-mediated inhibition of Fshβ transcription. This decrease in the FOXO1 deacetylation resulted in upregulation of Fshβ in rat primary pituitary cells and LβT2 cell line [76], further supporting the significance of SIRT1 in the HPG axis. Collectively, it was discovered that GnRH activation of Fshβ-expression was dependent on *miR-132/212*, which is dependent on the SIRT1-FOXO1 pathway.

The function of SIRT1 has also been discovered in the hypothalamic Kiss1 neurons, where it inhibits *Kiss1* activity [77]. Hence, SIRT1 controls puberty by regulating the puberty-stimulating gene, *Kiss1* [77]. In line with it, *Sirt1*-deficient mice exhibited central hypogonadism due to aberrant migration of GnRH neurons to the hypothalamus, suggesting that SIRT1 may play an important role in the regulation of the reproductive axis [78]. In addition, hypogonadotropic hypogonadism has been found in *Sirt1^−/−^* mice due to failure of GnRH neural migration. SIRT1’s catalytic domain promotes GN11 (mouse neuronal cell line) migration via deacetylating cortactin [78]. SIRT1 is found in the steroidogenic factor 1 (SF1) neuron of the ventromedial hypothalamic nucleus (VMH) and the pro-opiomelanocortin (POMC) and agouti-related protein (AgRP) neurons of the arcuate nucleus (ARH) [79,80,81]. Due to aberrant sympathetic activity, energy imbalance was seen in POMC neuron-specific *Sirt1^−/−^* mice [80]. Likewise, insulin resistance in skeletal muscles was observed in *Sirt1*-deficient SF1 neurons, while *Sirt1*-overexpression resulted in induced obesity and insulin resistance [79]. Moreover, overexpression of *Sirt1* prevented age-related weight gain in POMC or AgRP neurons. However, energy expenditure due to sympathetic activity was increased in the former one while food intake was reduced in the latter one, suggesting the existence of a hypothalamic nuclei-specific regulation [81]. Moreover, there was a higher level of SIRT1 in dorsomedial (DMH) and lateral hypothalamic nuclei (LHN) upon limiting the food provision [8]. Overexpression of *Sirt1* in the brain cells of mice resulted in a longer life span characterized by the overactivity of DMH and LHN via elevated levels of orexin type 2 receptor (Ox2r) [8], suggesting a tissue-specific role of SIRT1 in regulating and maintaining hunger, use of energy, metabolic activities, and longevity.

**Figure 6:**
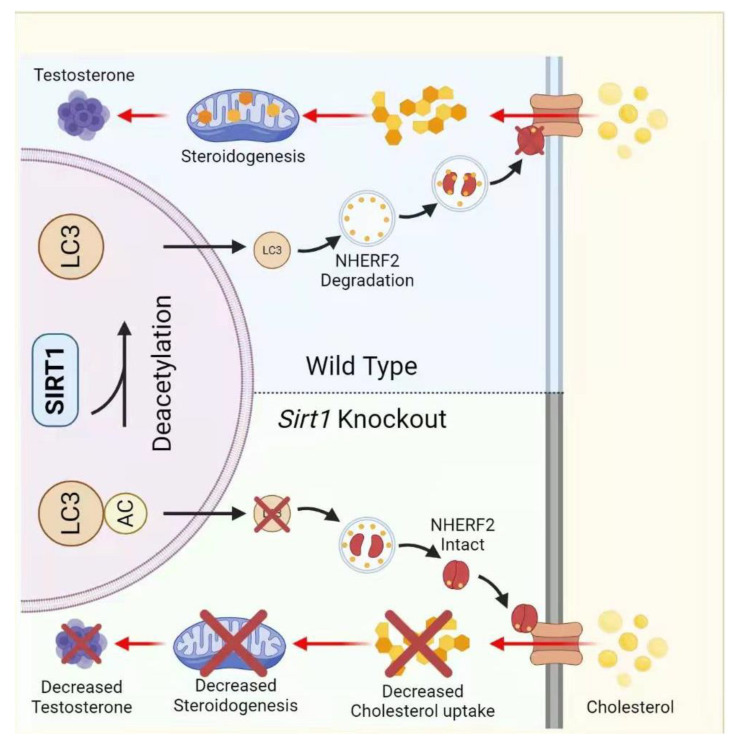
**Schematic cartoon of SIRT1-mediated cholesterol uptake in Leydig cells.** SIRT1 deacetylates the LC3 in the nucleus, which moves to the cytoplasm to participate in autophagosome biogenesis. The resulting autophagosomes are used to degrade NHERF2—thus allowing SR-BI to uptake cholesterol to fuel the process of testosterone biosynthesis.

*Sirt1*-knockdown results in low testosterone biosynthesis as it affects the Leydig cell maturation and reduces the steroidogenic acute regulatory protein (StAR) level [42]. Our group has recently reported that *Sirt1-*deficiency in the Leydig cells interferes with the cholesterol uptake due to compromised autophagy, and consequently results in a decreased testosterone biosynthesis in mice [82]. A steroidogenic cell-specific *Sirt1*-knockout mouse line was generated via mating *Sirt1^F/F^* mice with *SF1-Cre* strain (Figure 6). We found a significant decrease in testosterone levels and mating efficiency in *Sirt1^−/−^* mice. However, we found no differences in the testis size upon *Sirt1*-deletion. Furthermore, we observed that the mating efficiency instead of spermatogenesis was compromised in these mice (Figure 6) [65,82]. Finally, we figured out that, upon SIRT1-mediated deacetylation, LC3 moves from the nucleus to the cytoplasm and helps autophagosome formation, which degrades the NHERF2 (a negative regulator of cholesterol uptake receptor, scavenger receptor class B type I (SR-BI)). Consequently, it maintains the SR-BI level to uptake cholesterol, thus fueling the process of steroidogenesis. However, in *Sirt1^−/−^* mice, LC3 remains in the nucleus, inhibiting NHERF2 clearance, thus stopping SR-BI expression and cholesterol uptake, finally resulting in reduced testosterone biosynthesis.

Proinflammatory cytokines also have an important role in steroidogenesis [83]. SIRT1 has significant anti-inflammatory effects in the presence of cytokines [84]. *Sirt1* gene and protein levels in TNF-α-treated TM3 cells were found to be considerably lower, as were testicular *Sirt1* mRNA levels in high-fat-induced obese mice. A huge increase in the cytokines and decrease in the genes expression of several steroidogenic enzymes were observed in *Sirt1*-deficient TM3 cells. This boom in cytokine levels halts the transactivation of *SF1*. In contrast, *Sirt1*-overexpression enhances *SF1*-activity and consequently the steroidogenic enzymes and testosterone biosynthesis [84].

## 5. Conclusions and Prospects

Based on the information presented above, SIRT1 seems an important participant in male reproductive processes. Moreover, SIRT1 activation or depletion greatly affects male reproduction in various ways. In contrast to other sirtuins, researchers have extensively studied SIRT1, but its role in male reproduction still requires a lot of effort, as the exact molecular mechanisms of SIRT1 in regulating male reproduction have not been fully explored yet. Another issue is that most of the research thus far has been conducted using animal models, and little is known about the effects of SIRT1 on human male fertility.

Recently, we found that *Sirt6* is also required for spermatogenesis [85], but the underlying mechanism is still largely unknown. How about other sirtuins in male reproduction? Are there any relationships between these sirtuins during male reproduction? If there were, how they were evolved to deal with different male reproductive processes? Therefore, addressing these questions and other related issues is very important to prevent or develop new methods to treat some sirtuin family-related male infertility.

## Data Availability

Not applicable.
